# Analysis of Heavy Metal Pollution in Cultivated Land of Different Quality Grades in Yangtze River Delta of China

**DOI:** 10.3390/ijerph18189876

**Published:** 2021-09-19

**Authors:** Hua Wang, Wuyan Li, Congmou Zhu, Xiaobo Tang

**Affiliations:** 1 Zhejiang University of Finance and Economics Dongfang College, Haining 314408, China; wangh_df@163.com (H.W.); jasontxb@hotmail.com (X.T.); 2 School of Earth Sciences and Engineering, Hohai University, Nanjing 211110, China; 3 Zhejiang University of Finance & Economics, Hangzhou 310018, China; 4 Institute of Agriculture Remote Sensing and Information Technology, College of Environmental and Resource Sciences, Zhejiang University, Hangzhou 310058, China; congmouzhu1993@zju.edu.cn

**Keywords:** soil heavy metal, cultivated land quality grade, Nemerow integrated pollution index (NIPI), driving force

## Abstract

The distribution of heavy metal pollution in cultivated land is closely related to the quality of the cultivated land. In this study, 533 soil samples were collected from cultivated land in the Yangtze River delta region in China for Cd, Pb, and Hg analyses. Spatial statistical analysis was used to study the heavy metal pollution in the cultivated land, and the driving forces of heavy metal distribution in different cultivated land quality subdivisions were analyzed with GeogDetector. The conclusions are as follows: (1) Among the three heavy metals in the study area, the coefficient of variation of Cd is the largest, and that of Pb is the smallest. The proportion of Cd and Hg exceeding the standard value (the standard of level two in GB 15618—2018) is relatively large, both of which are 5%; (2) From the perspective of the spatial distribution of soil heavy metal pollution, only four counties (CX, HN, WY, and LH) were free of heavy metal pollution. Soil heavy metal pollution in AJ, SY, QJ, and DS counties is relatively serious, and the pollution may come from agricultural activities, manufacturing, and prevalent coastal shipping industries in these counties; (3) The heavy metal pollution levels of cultivated land with different quality levels are different. The high-quality cultivated land has no high contamination, while the medium and the general cultivated land both have high contamination. High contamination is related to Cd for medium and general cultivated lands, and to Hg in only general cultivated land; (4) The main driving factors of heavy metal concentration in cultivated soil were GDP, followed by soil organic matter, and pH. These results indicate that the spatial distribution of heavy metal concentration in cultivated soil was affected by the level of economic development, followed by the ecological environment, indicating that human activities had a critical impact on the ecological environment of cultivated land.

## 1. Introduction

Soil heavy metals, due to their nondegradable, mobile, and toxic properties [1,2], directly or indirectly threaten food security, ecosystems, and human health through the food chain [3,4]. In 2014, China’s Ministry of Environmental Protection and the Ministry of Land and Resources released the National Soil Pollution Investigation Bulletin, showing that nearly one-fifth of the country’s arable land is now polluted by heavy metals. Cadmium pollution is prominent in cultivated soil in China. According to the China Soil Environmental Quality Standard (0.3 mg·kg^−1^), the cadmium-exceeding rate of cultivated soil is as high as 7.0% [5]. Heavy metal pollution in cultivated soil has attracted more and more attention. Soil heavy metal pollution can reduce plant diversity [6], cause the pollution of rice and other crops [7], and affect the evolution of soil microorganisms and their related functional genes [8]. The concentration of heavy metals in soil is an important factor affecting the quality grading of cultivated land [9]. Heavy metal pollution has been considered in a large number of studies on the quality of cultivated land, and heavy metal pollution has become a decisive factor in the natural quality of cultivated land in some areas of China.

Soil heavy metal pollution is closely related to anthropogenic pollution sources [10,11,12], and anthropogenic activities, such as industrial manufacturing, transportation, agricultural production, garbage treatment, urban sewage, and automobile exhaust, causing heavy metals to accumulate in the soil [13,14,15]. According to the soil metal background value of the China Environmental Protection Administration, the common heavy metals in Chinese soil that exceed their background value are: chromium (Cr); lead (Pb); cadmium (Cd); mercury (Hg); and arsenic (As) [16]. The soil pH, fine particulate matter, and the proximity to polluting enterprises significantly influence the heavy metal pollution in soils [10]. Guo et al. (2021) discuss the spatial distribution of heavy metals, as well as the environmental risks, with regard to the farmland soil of Jinhua City, Zhejiang Province, and divide the safe utilization area of heavy metals in farmland soil. Their study found that the concentration of Zn in Jinhua was the highest, followed by Cr, and only a small part of the cultivated land was at risk of utilization [17]. Hu et al. (2020) found that the dominant sources of Cr, Cd, Hg, and As on the Hangzhou–Jiaxing–Huzhou Plain were soil parent materials, industrial activities, atmospheric deposition, and agricultural inputs, respectively [18]. Doabi et al. (2018) found that in Kermanshah province, Iran, the pollution of heavy metals in some areas may come from anthropogenic sources [19]. In Hanzhong, China, the average Cd concentrations in rice were slightly higher than the allowable threshold (0.20 mg kg^−1^), and the main pollution sources of heavy metals were agricultural activities (As, Cu, Cd), transportation emissions (Cu, Pb), coal combustion (Hg, As), and smelting activities (Zn, Pb, Cu) [20].

The common pollution indexes for studying the heavy metal concentrations in soil include: the enrichment factor [21,22,23], the geoaccumulation Index [22,24,25], the Nemerow pollution index [26,27,28], the potential ecological risk index [25,27], and the biogeochemical index [29]. Geostatistics has been proven to be one of the most effective methods for analyzing the spatial distribution of heavy metals in soil and for evaluating the quality of cultivated land. The concentrations of Cu, Cr, Ni, Zn, Pb, Cd, Hg, Mn, and Co in the soil of Shanghai are all higher than the background value of soil in China, and the concentration of Hg is the highest [30]. McGrath et al. (2004) conducted a geostatistical analysis and risk factor assessment of the Pb concentration in the soil of a silver mining area in Ireland that provided useful reference information for decisionmakers assessing the environmental soil quality [31]. Wu et al. (2008) used soil types as replication information to analyze the spatial variability of Cu, Zn, Pb, and Cd in a polluted area of Fuyang City, Zhejiang Province, by the geostatistical method, so as to improve the accuracy of its spatial prediction [32]. The combination of GIS and multivariate analysis to identify heavy metal sources in soil is also very effective [33]. Ha et al. (2014) used the Kriging method and GIS technology to interpolate the nonpoint sources of heavy metal pollution and analyze their distribution characteristics [34]. Multivariate statistical analyses [25,35], principal component analysis [34,36], hierarchical cluster analysis [24,37], and cluster analysis [21] are also widely used in soil heavy metal evaluation. Marrugo-Negrete et al. (2017) used multivariate statistical analysis, principal component analysis, and cluster analysis to show that soil pollution was mainly caused by agricultural activities along the Sinu River Basin, Colombia [38]. Ying et al. (2016) adopted the potential ecological risk index to investigate the ecological risk of heavy metal in the urban area soils of the coal mining city, Huainan, East China [39]. Huang et al. (2018) established a multiple regression model to simulate the soil heavy metal accumulation in Xiamen, China [35]. Some scholars have also applied the random forest model in the evaluation of heavy metal pollution to classify the monitoring sample points, predict the spatial distribution of heavy metals in the soil, and reveal the relative importance of different environmental factors in the prediction and decision-making structure [10]. Gope et al. (2017) used cluster analysis to divide the study into three regions (Industrial, Busy Traffic, and National Highway) to analyze the differences in pollution levels [21]. Alonso et al. (2020) used hierarchical cluster analysis to find that the distribution of total concentration is related to the continuous drainage of mine waste into the Suratá River [24]. The above research methods are indeed effective, but the research on the interaction between factors is insufficient.

GeogDetector is mainly used to detect the spatial heterogeneity of a phenomenon and its driving mechanism. In other words, it is used to detect the spatial heterogeneity of the dependent variable, and the extent to which the independent variable X explains the dependent variable Y [40]. The factor detection of GeogDetector can express similarities within the same region, and differences among regions. The model can quantitatively express the spatial stratification heterogeneity of the research object, mainly through the similarities and differences of the intra-variances and inter-layer variances after the partition. The interaction detection of GeogDetector can identify the interaction between different risk factors and assess whether the explanatory power of the dependent variable Y will be increased or weakened when the factors work together, or whether the effects of these factors on Y are independent of each other. Therefore, the GeogDetector model can better discover the driving factors affecting the distribution of heavy metals, and the interactions between the driving factors.

The Yangtze River Delta is one of the economically developed regions in China, and its soil heavy metal pollution problem is more prominent [41]. According to China’s first survey on soil pollution, 15% of farmland in the Yangtze River Delta is contaminated with heavy metals, with the worst pollution occurring in the border area between Zhejiang, Shanghai, and Jiangsu provinces. The Yangtze River Delta is polluted by heavy metals, persistent organic pollutants, and other toxic substances. From 2002 to 2012, the heavy metal pollution on the Hangzhou–Jiaxing–Huzhou Plain increased gradually [18]. Liu et al. (2021) found that the regions with high soil Hg concentration in China were located on the southeast coast, and the soil Hg concentration gradually decreased from southeast to northwest [42].

According to the level of economic development and topography, this paper selects 11 counties in the Yangtze River Delta as the research area. This paper will analyze the spatial characteristics of heavy metal pollution in the study area and reveal the spatial differences of heavy metal pollution in cultivated lands of different qualities. The study also analyzes the driving factors of heavy metal pollution of different quality farmland and discovers the interaction between the driving factors.

## 2. Materials and Methods

### 2.1. Study Area

The 11 counties selected as the study area are all from Zhejiang Province (30.5–31.5° N, 120–121° E), located on the southeast coast of China and the south wing of the Yangtze River Delta. Its land area is 105,500 km^2^, which is one of the smallest provinces in China [43]. The terrain of Zhejiang slopes from southwest to northeast in a step-shaped way. The southwest is dominated by mountains, the middle is dominated by hills, and the northeast is a low flat alluvial plain. Located in the middle of the subtropical zone, Zhejiang has a humid monsoon climate, moderate temperature, four distinct seasons, abundant sunlight, and abundant rainfall. The soil of Zhejiang Province is mainly yellow soil and red soil, accounting for more than 70% of the total area, mostly distributed in the hilly mountains with paddy soils in the plains and valleys, and salt and desalinized soils along the coast. The land use structure of Zhejiang province is known as “seven mountains, one water and two fields”, and the per capita cultivated land is less than half of the national per capita cultivated land. The Yangtze River Delta is the largest economic zone in China, so there is a close relationship between soil pollution and human activities in this region [44].

The counties in the study area have rich topographic features: Haining (HN) and Cixi (CX) have coastal plain topographic features; Anji (AJ) and Lin’an (LA) are hilly terrains; the terrain of Qujiang (QJ), Wuyi (WY), and Shengzhou (SZ) is mainly hilly and basin; Songyang’s (SY) terrain is mainly mountainous; Pingyang (PY) and Linhai (LH) are located in the eastern coastal area, in mainly mountainous and hilly terrain; and Daishan’s (DS) terrain is hilly island. The cultivated land area of each county, and its proportion to the total area of the county, are shown in Table 1.

### 2.2. Data Sampling

**Figure 1:**
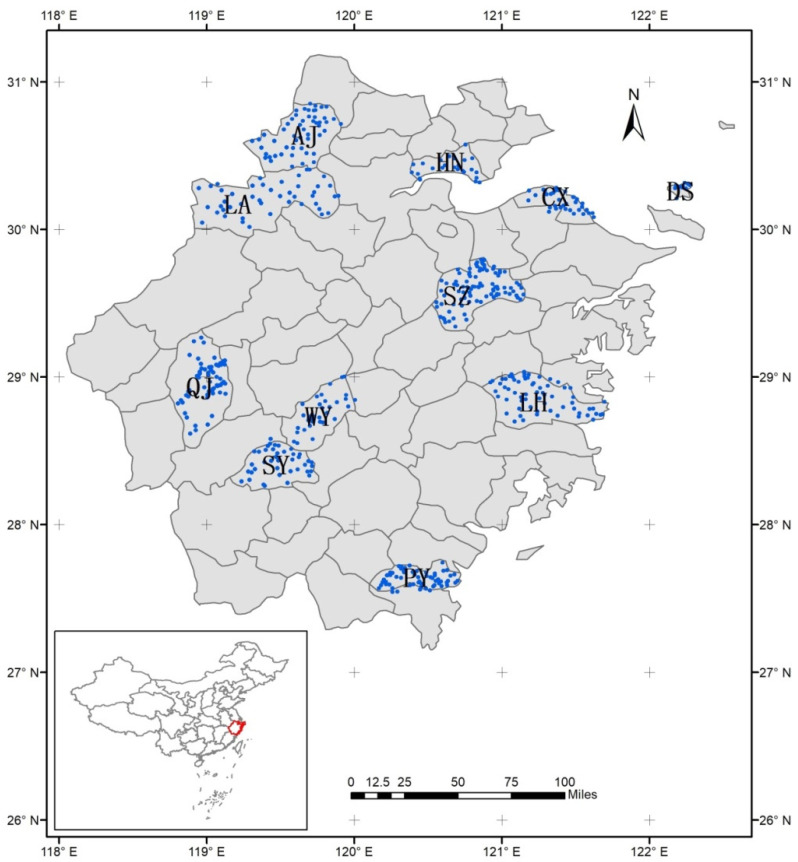
Distribution of sample points in the study area. The 11 counties with sample points are as follows: Lin ’an (LA), Anji (AJ), Haining (HN), Cixi (CX), Daishan (DS), Shengzhou (SZ), Qujiang (QJ), Wuyi (WY), Songyang (SY), Pingyang (PY) and Linhai (LH).

Based on the characteristics of topography, landform, and cultivated land quality in the study area, a total of 533 sample points were collected (Figure 1). Table 1 shows the number of sample points in each county. The specific sampling layout method is as follows [45]: Firstly, according to the mean square error and absolute deviation of the national natural land quality index, the sample size of the monitoring sampling points within the allowable error range was calculated; Secondly, the lag distance of the national natural land quality index was calculated and used as grid spacing to arrange points, so as to obtain the pre-layout results of monitoring sample points. Then, the monitoring sample sites were optimized according to the combination of different levels of cultivated land. Finally, according to the distribution of cultivated land quality, the classification of factors affecting the quality of cultivated land, the type of potential change of cultivated land, and its spatial distribution characteristics, the rationality of monitoring sample distribution was analyzed, and the accuracy of the sample distribution was evaluated by using the overall accuracy and the Kappa coefficient.

The cultivated land quality grade data came from the Ministry of Land and Resources of China [9].

### 2.3. Method for Measurement of Heavy Metal Concentration in Soils

We set a square of 1 m^2^ as a sampling unit and took 0–20 cm topsoil at its center point, and four corners within the unit, and selected 1.0 kg evenly mixed field samples as sampling samples. The sampling points were all positioned by GPS. All samples were air-dried at room temperature, crushed, and then thoroughly mixed with a 100-mesh nylon sieve for testing. The determination method of heavy metal concentration should refer to The Soil Environmental Quality Standard of China (GB 15618-1995). Cd and Pb were determined by graphite furnace atomic absorption spectrometry (GB/T 17141-1997), and Hg was determined by hydride generation atomic absorption spectrometry (HG-AAS). In the measurement, each sample was tested three times in parallel, and the relative error was controlled within 5%, and then its average value recorded. National standard soil samples were added in the analysis process for quality control to ensure that the test results were authentic.

### 2.4. Methods

#### 2.4.1. Assessment of Heavy Metal Pollution in Soils

The Nemerow integrated pollution index (NIPI) was used to calculate the single average pollution index (PI), and the comprehensive pollution index (I), of soil heavy metals [44]. The NIPI can only reflect the average pollution level of various pollutants in the soil, but also reflects the average pollution level of the most serious pollutants and can highlight the harm caused by them. The NIPI has been shown to be effective in quantifying the ecological and health risks of heavy metal pollution in agricultural soils [26]. The Nemerow integrated pollution index (NIPI) is calculated by Equation (2) [46,47,48].

(1) $$P_{i}=\frac{C_{i}}{S_{i}}$$

(2) $$\mathrm{NIPI}=\sqrt{\frac{P_{i}{}_{max}^{2}+P_{i}{}_{ave}^{2}}{2}}$$

where *Pi* is the pollution index of single heavy metal *i* in cultivated land; *Ci* is the concentration of heavy metal *i* (mg/kg); and *Si* is the evaluation standard of heavy metal *i* (mg/kg). The standard here adopts the Environmental Quality Evaluation Standards for Farmland of Edible Agricultural Products (Cd: 0.3, Pb: 50, Hg: 0.25). The higher *Pi* value, the more serious soil contamination is. According to the reference, soil heavy metal pollution can be divided into five levels: Uncontaminated, Warning Level of Caution, Low contamination, Moderate contamination, and High contamination (Table 2) [16,49,50,51].

#### 2.4.2. GeogDetector Model

The factor detection and interaction detection of the GeogDetector model can identify the driving factors of the soil heavy metal pollution of different cultivated land qualities and the interaction among the driving factors. The GeogDetector model can also test the spatial differentiation of a single variable and detect the possible causal relationship between two variables by testing the consistency of their spatial distribution. The model is as follows:

(3) $$q=1-\frac{\sum_{h=1}^{L}N_{h}\sigma_{h}^{2}}{N\sigma^{2}}$$

In Equation (3), q is the influence of factor X on heavy metal concentration Y; *h* = 1,…,*L* is the number of impact factors; $N_{h}$ is the number of partitions of the h-th factor; and N is the number of partitions of the entire region. $\sigma_{h}^{2}$ and $\sigma^{2}$ are the variance of the h-th factor and the heavy metal concentration Y in the whole region. The value range of *q* is between 0 and 1. When q = 0, it indicates that the spatial distribution of soil heavy metal concentration is not driven by influencing factors. When q = 1, it indicates that the spatial distribution of soil heavy metal concentration is strongly driven by influencing factors. The larger q value indicates the stronger explanatory power of each factor on the spatial distribution of soil heavy metal concentration.

## 3. Results and Discussion

### 3.1. Statistics of Soil Heavy Metals

Table 3 gives the descriptive statistics of Cd, Pb, and Hg in 533 soil samples. The mean concentration of the three heavy metals were 0.14 mg·kg^−1^, 28.47 mg·kg^−1^, and 0.14 mg·kg^−1^, respectively, which were in line with China’s environmental quality evaluation standards for farmland of edible agricultural products. Meanwhile, the median value of all heavy metals was lower than the average value. The coefficients of variation of the three heavy metals were 0.94, 0.65, and 0.72, respectively, among which Cd had the largest coefficient of variation, and Pb had the smallest coefficient of variation. The larger the coefficient of variation is, the more significant the spatial variation is, and this is easily affected by human activities.

Table 4 shows the background values and national soil quality standards for each county. According to the research of Wang Qinghua et al. [52], the background values of heavy metal in each county were determined. The county background value was taken as Grade I, the national environmental quality assessment standard for producing areas of edible agricultural products as Grade II, and the standard of level two in GB 15618-2018 as Grade III. From the mean value of heavy metal in each county, only AJ’s Cd exceeded the background value Grade I, and all the counties’ Cd did not exceed the standard value Grade II. In only two counties (PY and CX), Pb exceeded the background value (Grade I and Grade II); only DS’s Hg exceeded the background value (Grade I and Grade II).

For Cd, the average value of AJ and SY is relatively high, while the median value of LA and SY is relatively high. In terms of kurtosis and skewness, the distribution of Cd values in SZ, WY, and CX is positively skewed and lower than the normal distribution, while the other eight counties are positively skewed and higher than the normal distribution. The higher coefficients of variation were AJ (1.11) and SY (0.79). Pb, CX, and PY show the highest average and median concentrations. In terms of kurtosis and skewness, the distribution of Pb values in CX and WY is positively skewed and lower than the normal distribution, while the other nine counties and cities are positively skewed and higher than the normal distribution. QJ (0.97) and LH (0.67) had higher coefficients of variation. Hg, DS, and AJ show the highest average and median concentrations. In terms of kurtosis and skewness, the distribution of Hg values in AJ, LA, and DS is positively skewed and lower than the normal distribution, while the other eight counties are positively skewed and higher than the normal distribution. LH (0.78) and SY (0.74) had the highest coefficients of variation. In the total column, the degree for the coefficient of variation for Cd (0.94) is the largest among the three metals, and the proportion of Cd and Hg that exceed the standard value Grade III is relatively large (both 5%), indicating that these two heavy metals are greatly affected by human activities. In addition, the cultivated land surface soil heavy metal concentration in DS and AJ is slightly higher than other counties.

### 3.2. Spatial Differentiation of Heavy Metal Pollution in Cultivated Land

Table 5 shows the levels of heavy metal pollution in the cultivated land of 11 counties. Only four areas (CX, HN, WY and LH) are free of heavy metal pollution. Some sample points in SZ and QJ were at the Warning Level of Caution. AJ had the worst rating of all the counties, with 3.7% of the sample sites classified as moderately contaminated.

**Figure 2:**
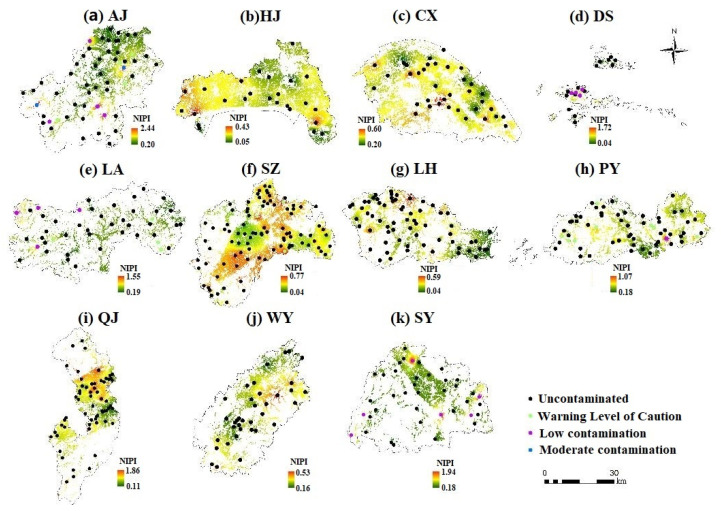
Spatial distribution of the cultivated land integrated pollution levels in 11 counties. Subfigures (**a**–**k**) are NIPI distribution maps of 11 counties respectively.

Figure 2 shows the spatial distribution of the integrated pollution levels of cultivated land in the study area, obtained by the inverse distance weighted interpolation method. It is found from the distribution map that AJ, SY, QJ, and DS have a larger maximum value of the NIPI, which indicates that the soil of these four counties is seriously polluted by heavy metals. The spatial distribution characteristics of AJ are that the pollution in the south is more serious than that in the north, and one or two sample sites in the north have slight pollution. The soil heavy metal pollution in the southeast and west of SY was more serious. In QJ, the pollution in the north is more serious, and the NIPI in the central area is the lowest. The agricultural land in QJ mainly consists of forest land, cultivated land, and garden plots. The pollution in QJ may be caused by the long-term excessive investment in chemical fertilizers and pesticides for agricultural activities, leading to the accumulation of heavy metal in the soil. Herbicides, pesticides, livestock and poultry manure, and municipal garbage also lead to the accumulation of heavy metal. The lacquer industry and animal husbandry are important industries in AJ, and their wastewater discharge pollutes the soil environment. The equipment manufacturing industry is an important economic pillar of AJ, QJ, and SY, and the dust produced in the production process pollutes the soil in the form of atmospheric deposition. The soil heavy metal pollution in DS is mainly concentrated in the southern islands. DS is located in the Zhoushan Islands, along the southeast coast of China. It is the hub of domestic north-south routes and river-sea-combined transportation. The maritime transportation industry is extremely developed. As the junction point of ocean transportation and water transportation, the busy shipping and the use of cargo collection and distribution transportation tools produces a large number of pollutants in the environment in this region, such as heavy metals and oils, which cause soil heavy metal pollution.

### 3.3. Characteristics of Heavy Metal Pollution in Cultivated Land under Different Land Use Types

Land use patterns can determine the distribution characteristics and driving factors of heavy metals in the soil [53].

Table 6 shows the concentration of heavy metals in cultivated soil under different land use types in the study area, including 160 dryland samples and 373 paddy fields samples. By means of comparison, it was found that the concentrations of the three heavy metals were lower than the background values (Grade II) in both dryland and paddy fields. The concentration of Cd and Hg in paddy fields were slightly higher than those in dryland, but the concentration of Pb in dryland was higher than that in paddy fields. The coefficients of variation of the three heavy metals in dryland and paddy fields were all greater than 0.6, among which the coefficient of variation of Cd was the largest, which was 0.973 and 0.928, respectively, followed by the variation degree of Hg in the dryland, which also reached 0.936.

According to the single factor pollution index, the NIPI of heavy metal pollution in dryland was Pb > Hg > Cd, and that of heavy metal pollution in paddy fields was Hg > Pb > Cd. In general, the average NIPI of paddy fields (0.532) was slightly higher than that of dryland (0.527), indicating that the heavy metal pollution of paddy fields was slightly more serious than that of dryland.

### 3.4. Characteristics of Heavy Metal Pollution in Cultivated Land of Different Quality Grades

According to the national cultivated land quality grading results released by the Ministry of Land and Resources of China, there are 15 grades of cultivated land in China, with Grade 1 being the best and Grade 15 being the worst. All the sample sites in the study area included nine cultivated land quality grades, ranging from 4 to 12. We set the classification standard according to the actual situation of the study area and the national classification standard (Announcement No.30, 2014 of the Ministry of Land and Resources). We classified Grade 4, Grade 5 and Grade 6 as high-quality cultivated land, Grade 7, Grade 8, and Grade 9 as medium cultivated land, Grade 11 and Grade 12 as general cultivated land, and we then evaluated the pollution of heavy metals in cultivated land.

Table 7 shows the number of sample points in each cultivated land quality grade. There was only one sample in the lowest grade and the highest grade, and there were many other samples in the middle grade, presenting a normal distribution. The concentration of Pb in cultivated land quality Grade 4 was the highest (71.6), which was located in CX. The mean value of Pb in the other grades was relatively small, and the lowest Pb concentration was 19.73 in Grade 11. Similarly, the highest Hg concentration was found in Grade 4, and the lowest Hg concentration was found in Grade 5 (0.11), with little difference in other cultivated lands. The highest Cd concentration was in Grade 10 (0.2), and the lowest was in Grade 4 and Grade 12 (0.04 for both).

**Figure 3:**
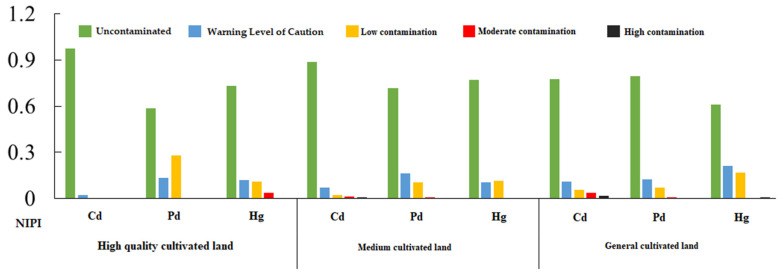
Heavy metal pollution index in different cultivated land quality zones.

Figure 3 shows the heavy metal NIPI in different cultivated land quality zones. In general, heavy metals in cultivated land pollution in the study area are uncontaminated, with more than 72.23% above the warning level of caution and some areas exceeding the standard. The heavy metal pollution levels of cultivated land with different quality grades are different. The proportion of soil heavy metals exceeding the background value (Grade II) was 6% in high-quality cultivated land, 7.32% in general cultivated land, and 14.45% in medium cultivated land. There was no high contamination in high-quality cultivated land, and the levels of low and moderate contamination were 5.44% and 0.56%, respectively. There is high contamination in medium cultivated land and general cultivated land. The low, moderate, and high contamination points in medium land are 12.57%, 1.5%, and 0.38%, respectively, and the three pollution points in general land are 5.82%, 0.94%, and 0.56%, respectively. High contamination is related to Cd for medium and general cultivated lands, and for Hg in only general cultivated land. The proportion of soil heavy metals exceeding the background value (Grade II) of Cd, Pb, and Hg were 5.07%, 13.32%, and 13.7%, respectively. In general, the high-quality cultivated land had no Cd pollution, the medium cultivated land had the most Pb and Hg pollution, and the general cultivated land had the most Hg pollution.

### 3.5. Driving Force Analysis of Heavy Metal Distribution

The GeogDetector model determines the influence of driving factors and explores the interaction and explanatory power of different driving factors on the concentration and distribution of heavy metals. Seven driving factors were selected in the study, among which the natural factors were pH, soil organic matter content, soil fertility, altitude and slope, and the human factors were gross domestic product (GDP) and the light index. The natural breaks method is used to discretize these continuous variables and make them into type variables. The GDP is based on 2018 GDP data from the townships where the monitoring sites are located. In this section, heavy metal pollution is still evaluated from three cultivated land quality zones: high-quality cultivated land, medium cultivated land, and general cultivated land.

Table 8 shows the analysis results of heavy metal driving force strength calculated by the GeogDetector model. The q statistics of each influencing factor, and the *p* value of the statistical test, are given in Table 8. The larger the q, the stronger the explanatory ability of this factor to heavy metals. The heavy metal concentrations of cultivated lands with different qualities were different in the driving intensity and direction of each index. For Cd, the GDP of high-quality cultivated land, the pH and soil organic matter content of medium cultivated land, and the pH of general cultivated land all passed the significance test, but their contributions to the spatial differentiation of Cd were not large enough. For Pb, GDP (0.39) has the largest contribution in high-quality cultivated land, followed by pH (0.36). GDP also has the largest contribution in medium cultivated land, and soil organic matter content is the largest contribution in general cultivated land. For Hg, soil organic matter content contributes the most in high-quality cultivated land, followed by GDP. The contribution of soil organic matter content to Hg was the largest in medium cultivated land, while GDP was the largest in general cultivated land.

In general, among the indicators that passed the significance test, the top three q values were GDP, soil organic matter content, and pH. The driving factors of soil heavy metal concentration in high-quality cultivated land were GDP, followed by soil organic matter content, and pH. The driving factors of soil heavy metal concentration in medium cultivated land were GDP and soil organic matter content. The driving factors of heavy metal concentration in general cultivated land were GDP, followed by pH, and soil organic matter content. These results indicate that the spatial differentiation of heavy metal concentration in cultivated soil is affected by the level of economic development, followed by the ecological environment. The results of the study once again prove that human activities, agriculture, and industrial production have an important impact on the ecological environments of cultivated land. 

In general, the concentration and spatial distribution of heavy metals in soil are affected by the interaction of multiple factors. Table 9, Table 10 and Table 11 shows the cross-influence intensity of the driving forces of heavy metals in soil. For Cd (Table 9), the cross-contribution between soil organic matter content and slope is the largest in high-quality cultivated land, followed by soil organic matter content, and pH. The cross-contribution of soil organic matter content and light index was the largest in medium cultivated land, followed by slope and pH, and the cross-contribution of pH and soil fertility was the largest in general cultivated land, followed by pH and organic matter content. For Pb (Table 10), the cross-contribution of pH—soil organic matter content is the largest in high-quality cultivated land, followed by pH—light index, and pH—GDP; the cross-contribution of pH—GDP is the largest in medium cultivated land, followed by GDP—altitude; the cross-contribution of pH—slope is the largest in general cultivated land, followed by GDP—altitude. For HG (Table 11), the cross-contribution of soil organic matter content—light index in high-quality cultivated land was the largest, followed by light index—pH. The cross-contribution degree of factors in medium cultivated land was uniform, and the cross-contribution degree of pH—light index, soil organic matter content—light index and pH—GDP were all 0.33. In general cultivated land, the cross-contribution of GDP—soil organic matter content is the largest, followed by GDP—pH.

It is worth noting that GDP is still the main contributing factor for most of the components affecting the interaction of soil heavy metal concentration, and the contribution degree after interaction is significantly higher than that of the individual driving factors. The driving force of Cd is mainly natural indexes, while Pb and Hg are more driven by human activities. In addition, the heavy metal concentration in high-quality cultivated land was mainly driven by natural indexes, while the heavy metal concentration in medium and general cultivated land was mainly driven by human activities and natural indexes. The results of this study may be related to the differences in soil physics and chemistry in different regions. In addition, from the perspective of interactive detection, the GeogDetector can explain the nonlinear relationship better than other correlation analyses.

## 4. Conclusions

In this paper, the Nemerow comprehensive pollution index method was used to evaluate the soil heavy metal pollution in the developed regions of China, and the GeogDetector was used to analyze the driving force of the concentration of heavy metals in the soil, and to elucidate the relationship between the quality of cultivated land and heavy metal pollution. The following conclusions were drawn:

(1) Among the three heavy metal concentration examined in the study areas, the coefficient of variation of Cd is the largest, and the coefficient of variation of Pb is the smallest. The samples of Cd and Hg exceeded the standard value (The standard of level two in GB 15618-2018) accounted for a large proportion. DS and AJ counties have slightly higher soil heavy metal concentration than other counties.

(2) Seen from the spatial distribution of heavy metal pollution levels, only four counties (CX, HN, WY and LH) are free of heavy metal pollution. Soil heavy metal pollution in AJ, SY, QJ, and DS is relatively serious, and the pollution may come from agricultural activities, manufacturing, and the coastal shipping industries in these counties.

(3) The heavy metal pollution of cultivated land with different types of land use showed that the concentrations of three heavy metals in dryland and paddy fields were lower than the background value. The concentration of Cd and Hg in paddy fields were slightly higher than those in dryland, but the concentration of Pb in dryland was higher than that in paddy fields. The average NIPI of paddy fields was slightly higher than that of dryland, indicating that the heavy metal pollution of paddy fields was slightly more serious than that of dryland.

(4) The heavy metal pollution levels of cultivated land with different quality levels are different. The high-quality cultivated land has no high contamination, while the medium and the general cultivated land both have high contamination. High contamination is related to Cd for medium and general cultivated lands, and for Hg in only general cultivated land.

(5) The main driving factors of soil heavy metal concentration in the study area were GDP, followed by soil organic matter content, and pH value. The results showed that the spatial distribution of heavy metal concentration in cultivated soil was affected by the level of economic development, followed by the influence of the ecological environment. It can be seen that human activities have an important impact on the ecological environment of cultivated land.

## Figures and Tables

**Table 1:** The cultivated land area and the number of sample points of each county.

Counties	Area of Cultivated Land (hm^2^)	Proportion of Cultivated Land to County Total Area (%)	The Number of Sample Points
LA	31,221.35	10.01	45
AJ	32,699.09	17.34	54
CX	45,764.20	34.63	36
HN	33,535.45	38.87	25
SZ	45,016.70	25.16	93
QJ	34,341.97	19.65	62
WY	24,359.13	15.53	36
LH	41,280.52	18.34	65
PY	31,370.91	30.10	60
SY	18,604.16	13.28	40
DS	4050.93	12.50	17

**Table 2:** Grading standard for heavy metal pollution degree of cultivated land.

Level	NIPI	The Degree of Contamination
1	Pontam	Uncontaminated
2	0.7 tyamin	Warning Level of Caution
3	1.0 ing 2.0	Low contamination
4	2.0 ntamin	Moderate contamination
5	Pco	High contamination

**Table 3:** Descriptive statistics of heavy metals in the study area.

Heavy Metal	Statistical Indicators	QJ	AJ	LH	LA	HN	SY	DS	SZ	CX	WY	PY	Total
Cd	Average (mg·kg^−1^)	0.11	0.24	0.08	0.20	0.10	0.23	0.21	0.13	0.08	0.09	0.11	0.14
	Median (mg·kg^−1^)	0.10	0.15	0.07	0.17	0.08	0.17	0.11	0.14	0.08	0.1	0.11	0.11
	Kurtosis	1.48	5.87	0.59	1.66	3.38	2.17	1.46	−1.02	−0.56	−0.78	0.29	22.58
	Skewness	1.08	2.51	0.89	1.35	1.57	1.82	1.76	0.08	0.25	0.12	0.32	4.09
	Coefficient of variation (%)	0.45	1.11	0.53	0.66	0.44	0.79	1.02	0.54	0.34	0.45	0.32	0.94
	Proportion above standard value (Grade III) (%)	0	0.17	0	0.18	0	0.15	0.18	0	0	0	0	0.05
Pb	Average (mg·kg^−1^)	24.17	24.76	19.13	24.95	5.8	38.74	28.66	20.54	57.87	18.51	52.19	28.47
	Median (mg·kg^−1^)	22.25	23.95	17.30	22.60	0.08	36.45	23.9	21	56.9	18.65	48.7	24.10
	Kurtosis	0.75	0.44	0.61	9.23	3.38	5.72	3.54	3.91	−0.26	−0.49	2.23	1.96
	Skewness	0.73	0.42	1.02	2.67	1.57	2.2	1.94	1.23	0.46	0.33	1.29	1.19
	Coefficient of variation (%)	0.97	0.39	0.67	0.37	0.37	0.44	0.45	0.62	0.17	0.45	0.37	0.65
	Proportion above standard value (Grade III) (%)	0	0	0	0	0	0	0	0	0	0	0	0
Hg	Average (mg·kg^−1^)	0.13	0.23	0.06	0.17	0.11	0.12	0.39	0.13	0.12	0.1	0.16	0.14
	Median (mg·kg^−1^)	0.11	0.23	0.05	0.15	0.09	0.09	0.29	0.11	0.11	0.1	0.15	0.12
	Kurtosis	0.25	−0.12	14.65	−0.62	2.8	1.18	−0.98	0.07	2.16	1.01	1.94	9.53
	Skewness	0.94	0.43	3.44	0.39	1.41	1.32	0.71	0.82	1.08	0.71	1.10	2.25
	Coefficient of variation (%)	0.17	0.32	0.78	0.50	0.68	0.74	0.63	0.55	0.39	50.29	0.48	0.72
	Proportion above standard value (Grade III) (%)	0.08	0.13	0	0	0	0	0.29	0.01	0	0	0.03	0.05

**Table 4:** Soil heavy-metal-related standards (mg·kg^−1^).

Standards		Cd	Pb	Hg
Background value (Grade I)	LA, AJ, HN	0.206	38.2	0.247
	CX, SZ, DS	0.213	53.1	0.232
	LH	0.232	42.2	0.181
	PY	0.253	51.2	0.251
	QJ, WY, SY	0.274	42.2	0.150
Environmental quality evaluation standards for farmland of edible agricultural products (Grade II)		0.3	50	0.25
The standard of level two in GB 15618-2018 (Grade III)		0.5	80	0.5

**Table 5:** Levels of heavy metal pollution in cultivated land in 11 counties.

Area	Uncontaminated	Warning Level of Caution	Low Contamination	Moderate Contamination
LA	82.22%	8.89%	8.89%	0.00%
AJ	81.48%	7.41%	7.41%	3.70%
CX	100.00%	0.00%	0.00%	0.00%
HN	100.00%	0.00%	0.00%	0.00%
SZ	96.77%	3.23%	0.00%	0.00%
QJ	98.39%	1.61%	0.00%	0.00%
WY	100.00%	0.00%	0.00%	0.00%
LH	100.00%	0.00%	0.00%	0.00%
PY	85.00%	13.33%	1.67%	0.00%
SY	82.50%	2.50%	15.00%	0.00%
DS	76.47%	0.00%	23.53%	0.00%

**Table 6:** Heavy soil metal pollution in different farmland types.

Farmland Types	Heavy Metal	The Average Heavy Metal Concentration (mg·kg^−1^)	The Average of the NIPI	Coefficient of Variation (%)
Dryland (n = 160)	Cd	0.126	0.42	0.973
	Pb	32.11	0.64	0.636
	Hg	0.131	0.52	0.936
Paddy field (n = 373)	Cd	0.146	0.486	0.928
	Pb	26.91	0.538	0.654
	Hg	0.146	0.584	0.629

**Table 7:** The number of sample points in each cultivated land quality grade.

Grades	4	5	6	7	8	9	10	11	12
The number of sample points	1	32	49	81	101	143	93	32	1

**Table 8:** Driving force strength of soil heavy metal concentration in typical areas.

Driving Factors	Cd			Pb			Hg		
	High	Medium	General	High	Medium	General	High	Medium	General
X_1_	0.17	0.05 *	0.17 *	0.36 ***	0.02	0.07	0.12	0.02	0.06
X_2_	0.13	0.05 *	0.06	0.21 *	0.04	0.15 **	0.43 ***	0.10 ***	0.12
X_3_	0.06	0.03	0.06	0.22 ***	0.01	0.05	0.03	0.03	0.04
X_4_	0.20	0.04	0.08	0.05	0.04	0.12	0.16	0.07 ***	0.11
X_5_	0.18	0.04	0.10	0.10	0.03	0.05	0.05	0.05 *	0.10
X_6_	0.05	0.03	0.12	0.14	0.03	0.08	0.05	0.06	0.12
X_7_	0.19 *	0.01	0.04	0.39 ***	0.12 ***	0.09	0.23 *	0.05 *	0.27 ***

Note: ***, ** and * indicate that the variable is significant at the level of 1%, 5%, and 10%, respectively. X_1_, X_2_, X_3_, X_4_, X_5_, X_6_, and X_7_ represent pH value, soil organic matter content, soil fertility, altitude, slope, light index, and GDP, respectively. High, Medium, and General represent the high-quality cultivated land, the medium cultivated land, and the general cultivated land, respectively.

**Table 9:** Driving force cross influence of Cd.

Cultivated Land Quality	Driving Factors	X_1_	X_2_	X_3_	X_4_	X_5_	X_6_	X_7_
High	X_1_	0.17						
	X_2_	0.74	0.13					
	X_3_	0.36	0.31	0.06				
	X_4_	0.55	0.53	0.28	0.20			
	X_5_	0.69	0.82	0.42	0.61	0.18		
	X_6_	0.61	0.66	0.23	0.50	0.67	0.05	
	X_7_	0.56	0.73	0.33	0.46	0.64	0.46	0.19
Medium	X_1_	0.05						
	X_2_	0.28	0.05					
	X_3_	0.13	0.12	0.03				
	X_4_	0.28	0.20	0.12	0.04			
	X_5_	0.30	0.24	0.10	0.18	0.04		
	X_6_	0.28	0.33	0.13	0.16	0.23	0.03	
	X_7_	0.26	0.18	0.12	0.16	0.25	0.18	0.01
General	X_1_	0.17						
	X_2_	0.53	0.06					
	X_3_	0.60	0.26	0.06				
	X_4_	0.48	0.32	0.39	0.08			
	X_5_	0.52	0.32	0.34	0.51	0.10		
	X_6_	0.51	0.47	0.30	0.37	0.45	0.12	
	X_7_	0.38	0.34	0.19	0.37	0.46	0.24	0.04

**Table 10:** Driving force cross influence of Pb.

Cultivated Land Quality	Driving Factors	X_1_	X_2_	X_3_	X_4_	X_5_	X_6_	X_7_
High	X_1_	0.36						
	X_2_	0.79	0.21					
	X_3_	0.56	0.50	0.22				
	X_4_	0.62	0.60	0.39	0.05			
	X_5_	0.74	0.62	0.49	0.40	0.10		
	X_6_	0.78	0.64	0.54	0.38	0.60	0.14	
	X_7_	0.78	0.70	0.54	0.55	0.66	0.59	0.39
Medium	X_1_	0.02						
	X_2_	0.28	0.04					
	X_3_	0.12	0.18	0.01				
	X_4_	0.21	0.21	0.14	0.04			
	X_5_	0.21	0.23	0.15	0.25	0.03		
	X_6_	0.17	0.18	0.13	0.20	0.21	0.03	
	X_7_	0.35	0.28	0.19	0.34	0.32	0.27	0.12
General	X_1_	0.07						
	X_2_	0.51	0.15					
	X_3_	0.27	0.36	0.05				
	X_4_	0.53	0.46	0.37	0.12			
	X_5_	0.57	0.45	0.21	0.56	0.05		
	X_6_	0.54	0.50	0.28	0.40	0.42	0.08	
	X_7_	0.51	0.50	0.33	0.56	0.53	0.46	0.09

**Table 11:** Driving force cross influence of Hg.

Cultivated Land Quality	Driving Factors	X_1_	X_2_	X_3_	X_4_	X_5_	X_6_	X_7_
High	X_1_	0.12						
	X_2_	0.73	0.43					
	X_3_	0.46	0.69	0.03				
	X_4_	0.60	0.76	0.25	0.16			
	X_5_	0.73	0.82	0.19	0.40	0.05		
	X_6_	0.88	0.90	0.35	0.56	0.53	0.05	
	X_7_	0.71	0.84	0.40	0.66	0.59	0.68	0.23
Medium	X_1_	0.02						
	X_2_	0.30	0.10					
	X_3_	0.12	0.19	0.03				
	X_4_	0.17	0.30	0.15	0.07			
	X_5_	0.22	0.27	0.12	0.25	0.05		
	X_6_	0.33	0.33	0.21	0.19	0.20	0.06	
	X_7_	0.33	0.28	0.17	0.21	0.25	0.25	0.05
General	X_1_	0.06						
	X_2_	0.48	0.12					
	X_3_	0.37	0.35	0.04				
	X_4_	0.43	0.40	0.35	0.11			
	X_5_	0.53	0.60	0.30	0.66	0.10		
	X_6_	0.44	0.43	0.36	0.31	0.49	0.12	
	X_7_	0.65	0.66	0.60	0.57	0.52	0.47	0.27

## Data Availability

The data presented in this study are available on reasonable request from the corresponding author. The data are not publicly available due to privacy or ethical considerations.
